# Association Between Chinese Herbal Medicine Therapy and the Risk of Chronic Kidney Disease in Gout Patients

**DOI:** 10.3389/fphar.2021.661282

**Published:** 2021-05-17

**Authors:** Yan-Zhuan Xiao, Zhi-Zhong Ye, Yuan-Tong Liang, Xin-Peng Chen, Yu-Hsun Wang, Qiang Xu, James Cheng-Chung Wei

**Affiliations:** ^1^ Department of Rheumatology, The First Affiliated Hospital of Guangzhou, University of Chinese Medicine, Guangzhou, China; ^2^ The First Clinical Medical School, Guangzhou University of Chinese Medicine, Guangzhou, China; ^3^ Department of Rheumatology, Shenzhen Futian Hospital for Rheumatic Diseases, Shenzhen, China; ^4^ Department of Medical Research, Chung Shan Medical University Hospital, Taichung, Taiwan; ^5^ Department of Allergy, Immunology and Rheumatology, Chung Shan Medical University Hospital, Taichung, Taiwan; ^6^ Institute of Medicine, Chung Shan Medical University, Taichung, Taiwan; ^7^ Graduate Institute of Integrated Medicine, China Medical University, Taichung, Taiwan

**Keywords:** gout, chronic kidney disease, pharmacologic (drug) therapy, treatment, Chinese herbal medicine

## Abstract

**Background:** Chinese herbal medicine (CHM) has been nationally and globally used in treating gout for over a millennium. The potential relationship between the incidence of chronic kidney disease (CKD) in gout patients and CHM therapy is unclear. Thus, this study aimed to provide some evidence regarding the relationship between CHM therapy and the occurrence of CKD in gout patients.

**Methods**: We used data from the National Health Insurance Research database (NHIRD) in Taiwan. In this population-based nested case-control study, all participants were identified by International Classification of Diseases, Ninth Revision (ICD-9). Conditional logistic regression was used to calculate the odds ratio (OR) of the risk of CKD in gout patients treated with CHM therapy.

**Results**: Data on 1718 gout patients with CKD and 1:1 matched 1718 gout patients without CKD were collected for analysis. The results showed that CHM therapy in gout patients did not increase the risk of developing CKD (adjusted OR = 1.01; 95% confidence interval [CI]: 0.86–1.18; *p* > 0.05). Moreover, CHM therapy in gout patients for >365 days did not increase the incidence of CKD (adjusted OR = 1.30; 95% CI: 0.90–1.88; *p* = 0.162).

**Conclusion**: Traditional CHM therapy does not increase the incidence of CKD in gout patients.

## Introduction

The incidence of gout is the highest among all types of arthritis (Kuo et al., 2015). According to the Global Health Data Exchange and the World Health Organization database, the global incidence and prevalence of gout have increased rapidly over the past 3 decades, with a 37 and 41% increase in its incidence and prevalence, respectively from 1992 to 2017 (Mattiuzz and Lippi, 2020). Clinical manifestations include severe joint pain, swelling, and elevated skin temperature during an acute attack as well as inflammation and tissue destruction caused by deposition of monosodium urate crystals in the joints and other tissues (Roddy and Choi, 2014). Previous studies have shown that gout is an independent risk factor for chronic kidney disease (CKD). In one study, 24% gout patients presented with CKD stage ≥3 (Roughley et al., 2015). A systematic review found that the risk of developing kidney disease in patients diagnosed with gout was three times higher than in patients not diagnosed with gout (Kuo et al., 2016). Furthermore, CKD often progresses to end-stage renal disease, which is associated with premature death in gout patients (Kuo and Luo, 2017).

The goal of gout treatment in the acute stage of gouty arthritis mainly focuses on relieving pain while slowing down or stopping progression and further gout flares in the chronic stage, ultimately improving joint function (Lu et al., 2020) and increasing the quality of life. Furthermore, it is important to reduce the incidence of gout-related CKD.

Drugs such as nonsteroidal anti-inflammatory drugs (NSAIDs), colchicine, and corticosteroids are recommended to alleviate severe pain during an acute gout attack. These drugs are effective for short-term management; however, their long-term use can cause adverse effects such as gastrointestinal reactions, rashes, and even renal failure (Smith et al., 2011; Perez-Ruiz et al., 2015; Smith et al., 2011; Perez-Ruiz et al., 2015). Moreover, such drugs cannot prevent or reverse the progression of this chronic metabolic disease. The current international guidelines recommend the use of xanthine oxidase inhibitors (such as allopurinol and febuxostat) as first-line treatment and uricosuric agents (such as benzbromarone) as second-line treatment for chronic gout. Nevertheless, allopurinol causes allergic reactions among Asians (Chen et al., 2019), febuxostat is associated with increased cardiovascular risk (Jutkowitz et al., 2014), and benzbromarone causes liver failure (Haring et al., 2013). Although several studies have been conducted on gout, the current treatment drugs are not satisfactory since all urate-lowering drugs have potential side effects and drug interactions. Therefore, there is an urgent need for clinical treatments or drugs with high safety and good efficacy in lowering the concentration of uric acid to prevent the occurrence and progression of gout.

Traditional Chinese medicine (TCM) has a unique theoretical system and has shown significant clinical efficacy in the treatment of gout. Over the past few decades, extensive clinical trials have been conducted, especially in China (Chen, 2014; Xiao et al., 2018; Chi et al., 2020), to evaluate the use of Chinese herbal medicine (CHM) in the treatment of gout. The basic principle of Chinese medicine is syndrome differentiation and treatment. Under this guidance, classical CHM formulas and agents, such as Simiao San, Modified Simiao San, Gout decoction, and Danxi Gout decoction, are widely used in the treatment of damp-heat syndrome in gout patients, achieving good clinical efficacy (Chen, 2014; Xiao et al., 2018; Chi et al., 2020). In a previous study comparing the therapeutic advantages of herbs used in CHM therapy and chemical drugs such as colchicine, etoricoxib, and celecoxib in gout treatment, CHM therapy showed better effect in relieving acute pain, reducing the rate of recurrence, and lowering the concentration of uric acid with fewer side effects (Chen, 2014; Xiao et al., 2018; Chi et al., 2020). Furthermore, (Chen, 2014; Xiao et al., 2018; Chi et al., 2020) found that a combination of CHM formulas and chemical drugs was better than the use of chemical drugs alone. However, there are safety concerns (such as hepatotoxicity, cardiotoxicity, and nephrotoxicity) with use of CHM therapy (Hong et al., 2006; Yang et al., 2007; Yang et al., 2012; Pan et al., 2020), severely hampering the clinical application and development of CHM therapies (Hong et al., 2006; Yang et al., 2007; Yang et al., 2012; Pan et al., 2020). Therefore, doctors and patients are seriously concerned that CHM therapy may increase the risk of CKD in gout treatment.

To our knowledge, no large-scale study has evaluated the association between CHM therapy and the incidence of CKD in gout patients. Thus, this study aimed to provide some evidence regarding the relationship between CHM therapy and the occurrence of CKD in gout patients.

## Methods

### Data Sources

**FIGURE 1:**
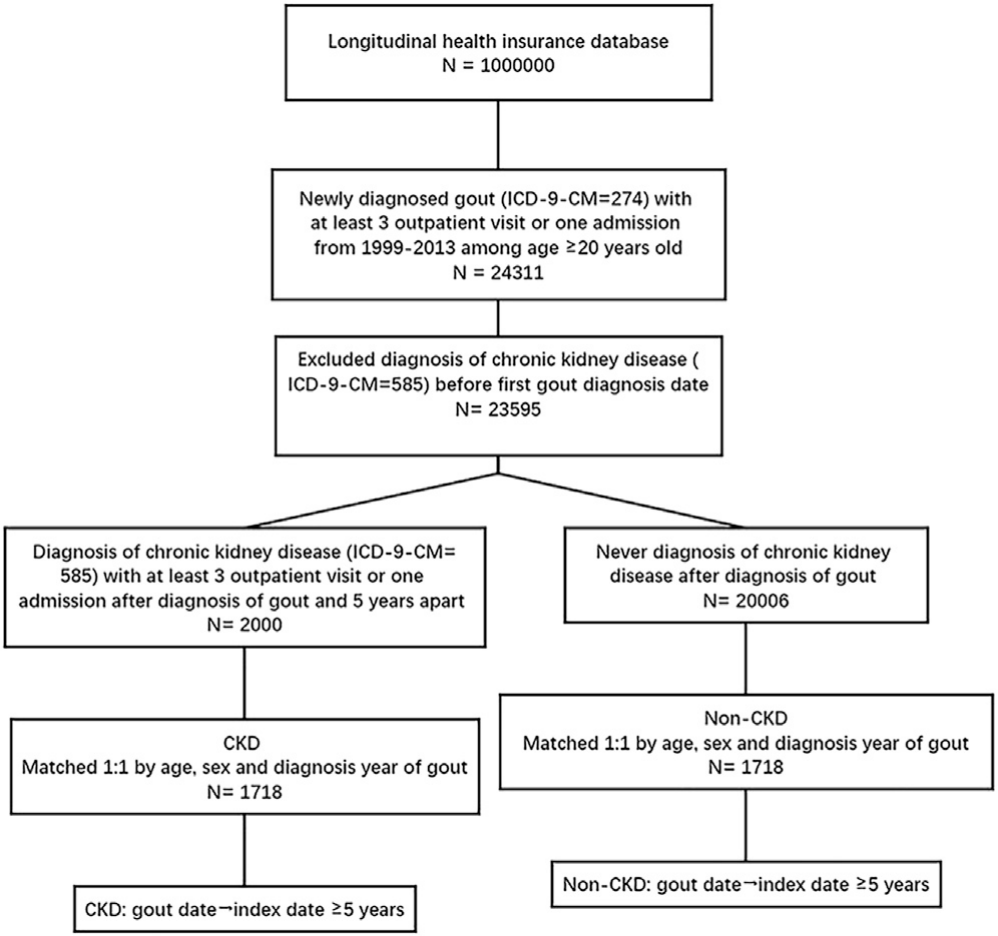
Flow chart of the study.

All research data were collected from the National Health Insurance Research database (NHIRD) of Taiwan from January 1, 1999 to December 31, 2013, and a total of One million people were randomly selected from the 23 million people included in the database Figure 1. The random sampling method was used by assigning serial numbers to 23 million beneficiaries in the sample population, and a random number generator was used to generate One million random values. To confirm the representativeness of the sample, we ensured that the age and sex distribution were not statistically different from that in the national sample. The NHIRD includes data on patient demographics, date of birth, sex, disease diagnoses, number of clinical visits and hospitalizations, and prescribed medications (with dosages), including CHM therapies administered by licensed TCM physicians. According to Taiwan Medical Law provisions, practicing physicians, including licensed TCM physicians, must be trained at a hospital or clinic designated by the central health authority for >2 years and obtain certification documents before commencing their individual practice. In Taiwan, licensed TCM physicians are required to make diagnoses using the International Classification of Diseases, Ninth Revision, Clinical Modification (ICD-9-CM) coding for claims. All diagnoses in this study were coded according to the ICD-9-CM.

### Participants

A total of One million subjects were randomly selected from the NHIRD. In total, 24,311 patients aged ≥20 years who were newly diagnosed with gout (ICD-9-CM code: 274) with least three outpatient visits or one hospitalization from January 1, 1999 to December 31, 2013 were selected in this study. Patients (*n* = 716) who had been diagnosed with CKD before the first diagnosis of gout were excluded.

The patients with gout with CKD were categorized into the CKD group, while those without CKD were categorized into the non-CKD group. The CKD group included 2,000 patients who were diagnosed with CKD (ICD-9-CM code: 585) with at least three outpatient visits or one hospitalization 5 years after the diagnosis of gout. The non-CKD group comprised 20,006 patients who had not been diagnosed with CKD within 5 years after the diagnosis of gout.

The index date was set at 5 years after diagnosis with gout for both groups. The ratio of patients in the CKD group to those in the non-CKD group was 1:1; all subjects were matched in terms of age, sex, and gout diagnosis year.

### Chinese Herbal Medicine and Covariates

The use of CHM was estimated from the first diagnosis of gout to the index date, and cumulative days of CHM therapy were used to calculate the dose effect. The main independent variables for CHM therapy among gout patients were selected from a series of demographic factors. Patients were classified into two groups according to age (<65 years and ≥65 years). Then, gout-associated diseases and treatments records were searched from the NHIRD database to identify independent variables. The baseline covariates included hypertension (ICD-9-CM codes: 401–405), hyperlipidemia (ICD-9-CM codes: 272.0–272.4), chronic liver disease (ICD-9-CM code: 571), diabetes (ICD-9-CM code: 250), chronic obstructive pulmonary disease (COPD; ICD-9-CM codes: 491, 492, and 496), autoimmune disease (ICD-9-CM codes: 710, 714, and 720), cardiovascular disease (ICD-9-CM codes: 410–414), and stroke (ICD-9-CM codes: 430–438). These covariates were identified based on at least three outpatient visits or one hospitalization for them between gout diagnosis and the index date. In addition, use of corticosteroids, statins, NSAIDs, and aspirin for a minimum of 30 days during the study period was evaluated.

### Statistical Analysis

Student’s t-test and the chi-square test were used to compare continuous and dichotomous variables, respectively, between CKD and non-CKD groups, and multivariate conditional logistic regression analysis was performed to investigate the risk of CKD in gout patients using CHM therapy after controlling for related confounding factors. SPSS version 18.0 (SPSS Inc, Chicago, IL, United States) was used for statistical analysis, and a P value of <0.05 was considered statistically significant.

## Results

### Demographic Characteristics of Gout Patients in the Chronic Kidney Disease and Non-Chronic Kidney Disease Groups

The demographic characteristics of gout patients in the CKD and non-CKD groups are shown in Table 1; the baseline characteristics were balanced between the groups. A total of 3,436 newly diagnosed gout patients were recruited from the claims database, including 1,718 patients in the CKD group and 1,718 patients in the non-CKD groups. The average age of the participants was approximately 68.3 years, with 34.7% patients aged 20–64 years and the remaining 65.3% patients aged ≥65 years. Among the enrolled patients, there were 27.5% female patients and 72.5% male patients. A total of 61.1 and 61.9% patients in the CKD and non-CKD groups were treated with CHM, respectively.

**TABLE 1:** Demographic characteristics of CKD and non-CKD.

	CKD (*N* =1718)		Non-CKD (*N* =1,718)		
	*n*	%	*n*	%	*p* value
Age					1
20–64	597	34.7	597	34.7	
≥65	1121	65.3	1121	65.3	
Mean ± SD	68.3 ± 12.2		68.3 ± 12.2		1
Sex					1
Female	472	27.5	472	27.5	
Male	1246	72.5	1246	72.5	
Hypertension	1480	86.1	1201	69.9	**<0.001**
Hyperlipidemia	952	55.4	843	49.1	**<0.001**
Chronic liver disease	545	31.7	490	28.5	0.041
Diabetes	797	46.4	560	32.6	**<0.001**
COPD	469	27.3	387	22.5	**0.001**
Autoimmune disease	170	9.9	163	9.5	0.686
Cardiovascular disease	725	42.2	616	35.9	**<0.001**
Stroke	479	27.9	341	19.8	**<0.001**
Traditional Chinese medicine	1049	61.1	1064	61.9	0.599
Corticosteroids	907	52.8	668	38.9	**<0.001**
Statin	825	48.0	598	34.8	**<0.001**
NSAIDs	1634	95.1	1620	94.3	0.286
Aspirin	925	53.8	752	43.8	**<0.001**
Gout year					**1**
2000	895	52.1	895	52.1	
2001	403	23.5	403	23.5	
2002	296	17.2	296	17.2	
2003	124	7.2	124	7.2	

Bold font indicates statistical significance (*p* < 0.05).

CKD, Chronic kidney disease; COPD, Chronic obstructive pulmonary disease; NSAIDs, Nonsteroidal anti-inflammatory drugs.

In this study, the incidence of hypertension, hyperlipidemia, chronic liver disease, diabetes, COPD, cardiovascular disease, and stroke was significantly higher in the CKD group than in the non-CKD group. Furthermore, patients in the CKD group received more corticosteroids, statins, and aspirin than those in the non-CKD group. The proportion of patients with hypertension (86.1 vs. 69.9%), hyperlipidemia (55.4 vs. 49.1%), chronic liver disease (31.7 vs. 28.5%), diabetes (46.4 vs. 32.6%), COPD (27.3 vs. 22.5%), cardiovascular disease (42.2 vs. 35.9%), and stroke (27.9 vs. 19.8%) was higher in the CKD group than in the non-CKD group (Table 1). Moreover, patients in the CKD group received more glucocorticoids (52.8 vs. 38.9%), statins (48.0 vs. 34.8%), and aspirin (53.8 vs. 43.8%) than those in CKD group. However, there was no significant difference in the proportion of patients with autoimmune disease between the groups. Similarly, there was no significant difference in the proportion patients receiving CHM treatment between the groups.

### Chinese Herbal Medicine Use did Not Increase the Risk of Developing Chronic Kidney Disease

Conditional logistic regression analysis was used to assess the risk factors of CKD, including covariates such as CHM use; common complications such as hypertension, hyperlipidemia, chronic liver disease, and diabetes, and some chemical drugs. At the 5 years follow-up, CHM use did not increase the risk of developing CKD (Table 2) since there was no relationship between the incidence of CKD in gout patients and CHM use (adjusted odds ratio [OR] = 1.01; 95% confidence interval [CI]: 0.86–1.18; *p* = 0.920).

**TABLE 2:** Conditional logistic regression of risk of CKD (1).

	Crude OR	95% C.I.	*p* value	Adjusted OR^a^	95% C.I.	*p* value
CHM	0.96	0.84–1.11	0.593	1.01	0.86–1.18	0.920
Hypertension	3.04	2.50–3.68	<0.001	2.50	2.03–3.09	**<0.001**
Hyperlipidemia	1.30	1.13–1.49	<0.001	0.84	0.70–0.998	**0.047**
Chronic liver disease	1.17	1.01–1.36	0.037	1.09	0.92–1.28	0.308
Diabetes	1.83	1.59–2.12	<0.001	1.48	1.26–1.73	**<0.001**
COPD	1.32	1.12–1.55	0.001	1.02	0.85–1.22	0.810
Autoimmune disease	1.05	0.84–1.31	0.690	0.98	0.77–1.25	0.862
Cardiovascular disease	1.33	1.15–1.54	<0.001	0.96	0.81–1.14	0.665
Stroke	1.58	1.34–1.85	<0.001	1.24	1.04–1.49	**0.019**
Corticosteroids	1.75	1.52–2.01	<0.001	1.68	1.44–1.95	**<0.001**
Statin	1.78	1.54–2.05	<0.001	1.49	1.24–1.79	**<0.001**
NSAIDs	1.18	0.87–1.59	0.286	0.96	0.69–1.33	0.790
Aspirin	1.58	1.37–1.83	<0.001	1.06	0.89–1.27	0.504

Bold font indicates statistical significance (*p* < 0.05).

CHM, Chinese herb medicine; COPD, Chronic obstructive pulmonary disease; NSAIDs, Nonsteroidal anti-inflammatory drugs.

a Adjusted for hypertension, hyperlipidemia, chronic liver disease, diabetes, COPD, autoimmune disease, cardiovascular disease, stroke, corticosteroids, statin, NSAIDs, and aspirin.

Hypertension (adjusted OR = 2.50; 95% CI: 2.03–3.09; *P* < 0.001), diabetes (adjusted OR = 1.48; 95% CI: 1.26–1.73; *P* < 0.001), and stroke (adjusted OR = 1.24; 95% CI: 1.04–1.49; *P* = 0.019) were associated with a higher risk of developing CKD. However, hyperlipidemia (adjusted OR = 0.84; 95% CI: 0.70–0.998; *P* = 0.047), chronic liver disease (adjusted OR = 1.09; 95% CI: 0.92–1.28; *P* = 0.308), COPD (adjusted OR = 1.02; 95% CI: 0.85–1.22; *P* = 0.810), autoimmune disease (adjusted OR = 0.98; 95% CI: 0.77–1.25; *P* = 0.862), and cardiovascular disease (adjusted OR = 0.96; 95% CI: 0.81–1.14; *P* = 0.665) did not increase the risk of developing CKD. Furthermore, the use of corticosteroid (adjusted OR = 1.68; 95% CI: 1.44–1.95; *P* < 0.001) and statin (adjusted OR = 1.49; 95% CI: 1.24–1.79; *P* < 0.001) increased the risk of CKD, whereas the use of NSAIDs (adjusted OR = 0.96; 95% CI: 0.69–1.33; *P* = 0.790) and aspirin (adjusted OR = 1.06; 95% CI: 0.89–1.27; *P* = 0.504) did not increase the risk of CKD.

### Chinese Herbal Medicine Use did Not Increase the Risk of Developing Chronic Kidney Disease in Different Sub-Populations

Conditional logistic regression analysis was used to evaluate the association between CHM use and the risk of developing CKD in gout patients according to age and sex. Further stratification analysis according to different age groups (<65 years vs. ≥65 years) and sex (female vs. male) showed that there was no significant difference between the sub-populations (Table 3).

**TABLE 3:** Conditional logistic regression of risk of CKD (2).

	*N*	No. of CKD	Crude OR	95% C.I.	*p* value	Adjusted OR^a^	95% C.I.	*p* value
Age <65								
CHM								
No	417	217	1			1		
Yes	777	380	0.88	0.69–1.12	0.297	1.01	0.77–1.34	0.923
Age ≥65								
CHM								
No	906	452	1			1		
Yes	1336	669	1.01	0.85–1.2	0.930	1.03	0.85–1.24	0.783
Female								
CHM								
No	292	149	1			1		
Yes	652	323	0.94	0.70–1.25	0.658	1.04	0.75–1.45	0.801
Male								
CHM								
No	1031	520	1			1		
Yes	1461	726	0.97	0.83–1.14	0.714	1.00	0.84–1.19	0.997

CHM, Chinese herb medicine; CKD: Chronic kidney disease.

a Adjusted for hypertension, hyperlipidemia, chronic liver disease, diabetes, COPD, autoimmune disease, cardiovascular disease, stroke, corticosteroids, statin, NSAIDs, and aspirin.

As shown in Table 3, the age of 1,194 and 2,242 patients was <65 and ≥65 years, respectively. Of the 777 patients aged <65 years who received CHM therapy, 380 patients did not develop CKD (adjusted OR = 1.01; 95% CI: 0.77–1.34; *P* < 0.05). However, of the 1,336 patients aged ≥65 years who received CHM therapy, 669 patients did not develop CKD (adjusted OR = 1.03; 95% CI: 0.85–1.24; *P* < 0.05).

Moreover, of the 652 female patients who received CHM therapy, 323 patients did not develop CKD (adjusted OR = 1.04; 95% CI: 0.75–1.45; *P* < 0.05), and of the 1,461 male patients who received CHM therapy, 726 patients did not develop CKD (adjusted OR = 1.00; 95% CI: 0.84–1.19; *P* < 0.05).

### Long-Term Use of Chinese Herbal Medicine did Not Increase the Incidence of Chronic Kidney Disease in Gout Patients

Conditional logistic regression analysis did not reveal an association between cumulative days of CHM therapy (<90 days, 90–180 days, 180–365 days, ≥365 days) and the incidence of CKD in gout patients (Table 4) even after ≥365 days (adjusted OR = 1.30; 95% CI: 0.90–1.88).

**TABLE 4:** Conditional logistic regression of risk of CKD (3).

	*N*	No. of CKD	Crude OR	95% C.I.	*p*-value	Adjusted OR^a^	95% C.I.	*p*-value
Cumulative days of CHM								
None	1323	669	1			1		
<90 days	977	476	0.92	0.79–1.07	0.259	0.98	0.83–1.16	0.821
90–180 days	338	157	0.90	0.68–1.19	0.456	0.84	0.62–1.14	0.266
180–365 days	798	416	1.33	0.98–1.8	0.069	1.28	0.92–1.79	0.143
≥365 days	3436	1718	1.17	0.83–1.64	0.377	1.30	0.90–1.88	0.162

CHM, Chinese herb medicine; CKD, Chronic kidney disease.

a Adjusted for hypertension, hyperlipidemia, chronic liver disease, diabetes, COPD, autoimmune disease, cardiovascular disease, stroke, corticosteroids, statin, NSAIDs, and aspirin.

### Top 10 Used Chinese Herbal Medicine Formulas

Table 5 shows the top 10 used CHM formulas used in this study. They were Shujing Huoxue decoction (3.6%), Duhuo Jisheng decoction (2.4%), Jisheng Shenqi pill (2.2%), Shaoyao Gancao decoction, Danggui Niantong decoction, Xuefu Zhuyu decoction, Liuwei Dihuang pill, Ganlu Yin, Jiawei Xiaoyao powder, and Chuanxiong Chatiao powder. Besides, the compositions of the top 20 formulas have been put in the supplementary document (Supplementary Table S1).

**TABLE 5:** The top 20 used Chinese medicine formulas.

Chinese medicine formula	Frequency	%
Shujing Huoxue Decoction	2574	3.6
Duhuo Jisheng Decoction	1744	2.4
Jisheng Shenqi Pill	1576	2.2
Shaoyao Gancao Decoction	1418	2.0
Danggui Niantong Decoction	1332	1.9
Xuefu Zhuyu Decoction	1275	1.8
Liuwei Dihuang Pill	1188	1.7
Ganlu Yin	1103	1.5
Jiawei Xiaoyao Powder	1073	1.5
Chuanxiong Chatiao Powder	1057	1.5
Gegen Decoction	1055	1.5
Yinqiao Powder	1043	1.5
Maxing Shigan Decoction	989	1.4
Tianwang Buxin Dan	981	1.4
Longdan Xiegan Decoction	981	1.4
Banxia xiexin Decoction	978	1.4
Pingwei Powder (Pill)	954	1.3
Zhibai Dihuang Pill	933	1.3
MaziRen Pill	930	1.3
Zhigancao Decoction	923	1.3

## Discussion

To our knowledge, this is the first study to investigate the correlation between the use of CHM and the risk of developing CKD in gout patients. The results showed that there was no association between CHM use and the occurrence of CKD gout patients.

Gouty arthritis is a chronic metabolic disease caused by abnormal purine metabolism and elevated serum uric acid concentrations, resulting in the deposition of urate crystals in the joints, kidneys, and other tissues (Lu et al., 2014). Therefore, patients with gout usually have comorbidities such as CKD. According to the United States National Health and Nutrition Examination Survey, the incidence of CKD of stage ≥2 in gout patients was 71% (Zhu et al., 2012). In a previous study, 24% gout patients had CKD stage ≥3 (Hong et al., 2006; Yang et al., 2007; Yang et al., 2012; Pan et al., 2020). Furthermore, according to a large population-based cohort study in the United Kingdom, compared to subjects without gout, those with gout had a 78% higher risk of developing CKD stage ≥3 within 3 years of diagnosis (Roughley et al., 2018). Furthermore, CKD is expected to become one of the five main causes of death by 2040, with enhanced risks of progression to end-stage renal disease and cardiovascular mortality (Foreman et al., 2018; Ortiz et al., 2019). Therefore, reducing the incidence of CKD or slowing down its progression in gout patients should be considered more seriously.

In this study, the incidence of hypertension, chronic liver disease, diabetes, COPD, cardiovascular disease, and stroke was significantly higher in the CKD group than in the non-CKD group. These diseases may be part of a series of clinical syndromes caused by impaired renal function, including metabolic syndrome and electrolyte imbalance. As established, the kidney is a significant target organ of serum urate; it has important endocrine and metabolic functions. Therefore, patients with hypertension, diabetes, or stroke have a higher risk of developing CKD than other individuals. Hypertension, stroke, and other diseases may cause abnormal vascular function and renal perfusion, resulting in vascular renal damage, while metabolic disorders such as hyperlipidemia and diabetes can further aggravate the burden of kidney function (Jankowski et al., 2021). In addition, corticosteroid use increases the risk of developing CKD, possibly due to water and sodium retention. Further, statins are excreted via the kidney, possibly increasing he incidence of adverse events. According to a large registry study, patients with cardiovascular diseases and CKD who receive fewer evidence-based therapies, such as statins, β-blockers, and antiplatelet medicine, may have higher mortality rates than other individuals (Washam et al., 2015).

In this study, the presence of hyperlipidemia appeared to be a protective factor against the risk of developing CKD among gout patients; this finding is contrary to that reported by previous studies. Some patients may have been diagnosed with hyperlipidemia and treated with lipid-lowering drugs for a short time, resulting in statistical error. However, the underlying mechanisms are unknown; hence, further studied are needed.

This population-based nested case-control cohort study, we clarified that our previous speculation that CHM therapy may harm the kidney was false, even in different age and sex sub-populations. CHM therapy has been used for the treatment of gout for many years. In recent years, CHM formulas are being modified or combined with chemical drugs to create gout regimens with fewer side effects, some of which have been regarded as promising treatments. Moreover, this study found that some Chinese medicine formulas have been widely used in gout patients, such as Shujing Huoxue decoction, Duhuo Jisheng decoction, and Jisheng Shenqi pill. Further, Simiao powder and modified CHM formulas such as Jiawei Simiao powder, Tongfeng decoction, and Danxi Tongfeng decoction are widely used for treating damp-heat syndrome in gout patients. Chen (2014) found that the TCM formulas combined with chemical drugs showed better effects than chemical drugs alone, a finding similar to that reported by Huang et al. (2018). Therefore, CHM might be a promising safe therapy in treating gout.

In addition, CHM can also be used to treat a variety of kidney diseases. In recent years, an increasing number of scholars have conducted relevant studies on the efficacy and safety of CHM therapy in the treatment of acute kidney injury, chronic renal insufficiency, chronic glomerulonephritis, and other kidney diseases. In a study by Kuo-Chin Huang, CKD patients treated with TCM exhibited an increased long-term survival rate. Shen et al. (2018) reported that CHM formulas combine different herbal compounds to increase or promote therapeutic effectiveness, minimize toxicity and side effects, and optimize the therapeutic effects of each component, providing a safe and effective therapy for renal fibrosis. Therefore, the current study does not support that CHM therapy for gout can increase the risk of developing CKD. However, CHM therapy may delay disease progression in gout patients with CKD and may prolong the onset of CKD in gout patients without CKD.

## Conclusion

This nested case-control study revealed that CHM therapy does not increase the incidence of CKD in gout patients, which clarifies our previous conjecture that traditional CHM may cause kidney damage. Further prospective clinical trials are needed to confirm our results.

## Data Availability

The raw data supporting the conclusion of this article will be made available by the authors, without undue reservation, to any qualified researcher.
