# Supplementary material for: Association Between Chinese Herbal Medicine Therapy and the Risk of Chronic Kidney Disease in Gout Patients
Source: Front Pharmacol. 2021 May 17;12:661282. doi: 10.3389/fphar.2021.661282 (PMC8165605; doi:10.3389/fphar.2021.661282)
Supplement: Supplementary file 1 [file table1.docx]

**Table S1. Composition of the top 20 used Chinese medicine formulas**

| **CHM Formula** | **Composition** | |
| --- | --- | --- |
|  | **Name** | **Family** |
| ShujingHuoxue Decoction | Danggui (Angelica sinensis)  Shaoyao (Paeonia lactiflora)  Shengdihuang (Rehmannia glutinosa)  Cangzhu (Atractylodes lancea)  Niuxi (Achyranthes bidentata)  Chenpi (Citrus reticulata)  Taoren (Persicae Semen)  Weilingxian (Clematis chinensis)  Chuanxiong (Ligusticum sinense)  Fangji (Stephania tetrandra)  Qianghuo (Notopterygium incisum)  Fangfeng (Saposhnikovia divaricata)  Baizhi (Angelica dahurica)  Longdancao (Gentiana scabra)  Fuling (Poriacocos)  Gancao (Glycyrrhiza uralensis) | Apiaceae  Paeoniaceae  Orobanchaceae  Asteraceae  Amaranthaceae  Rutaceae  Rosaceae  Ranunculaceae  Apiaceae  Menispermaceae  Apiaceae  Apiaceae  Apiaceae  Gentianaceae  Polyporaceae  Fabaceae |
| DuhuoJisheng Decoction | Duhuo (Heracleum hemsleyanum)  Sangjisheng (Taxillus sutchuenensis)  Fanfeng (Saposhnikovia divaricata)  Rougui (Cinnamomum cassia)  Duzhong (Eucommia ulmoides)  Niuxi (Achyranthes bidentata)  Xixin (Asarum heterotropoides)  Qingjiao (Gentiana macrophylla)  Fuling (Poriacocos)  Chuanxiong (Ligusticum sinense)  Renshen (Panax ginseng)  Danggui (Angelica sinensis)  Gancao (Glycyrrhiza uralensis)  Shaoyao (Paeonia lactiflora)  Shengdihuang (Rehmannia glutinosa) | Apiaceae  Loranthaceae  Apiaceae  Lauraceae  Eucommiaceae  Amaranthaceae  Aristolochiaceae  Gentianaceae  Polyporaceae  Apiaceae  Araliaceae  Apiaceae  Fabaceae  Paeoniaceae  Orobanchaceae |
| JishengShenqi Pill | Rougui (Cinnamomum cassia)  Fuzi (Aconitum carmichaeli)  Niuxi (Achyranthes bidentata)  Shudihuang ((Rehmannia glutinosa)  Shanyurou (Tetradium ruticarpum)  Shanyao (Dioscorea polystachya)  Fuling (Poria cocos)  Zexie (Alisma plantago-aquatica)  Cheqianzi (seed of Plantago asiatica)  Mudanpi (Paeonia suffruticosa) | Lauraceae  Ranunculaceae  Amaranthaceae  Orobanchaceae  Rutaceae  Dioscoreaceae  Polyporaceae  Alismataceae  Plantaginaceae  Paeoniaceae |
| ShaoyaoGancao Decoction | Shaoyao (Paeonia lactiflora)  Gancao (Glycyrrhiza uralensis) | Paeoniaceae  Fabaceae |
| DangguiNiantong Decoction | Qianghuo (Notopterygium incisum)  Gancao (Glycyrrhiza uralensis)  Yinchen (Artemisia capillaris)  Fanfeng (Saposhnikovia divaricata  Cangzhu (Atractylodes lancea)  Danggui (Angelica sinensis)  Zhimu (Anemarrhena asphodeloides)  Zhuling (Agaric Polyporus)  Zexie (Alisma plantago-aquatica)  Shengma (Cimicifuga foetida)  Baizhu (Atractylodes macrocephala)  Huangqin (Scutellaria baicalensis)  Gegen (Pueraria edulis)  Renshen (Panax ginseng)  Kushen (Sophora flavescens) | Apiaceae  Fabaceae  Asteraceae  Apiaceae  Asteraceae  Apiaceae  Asparagaceae  Polyporaceae  Alismataceae  Ranunculaceae  Asteraceae  Lamiaceae  Fabaceae  Araliaceae  Fabaceae |
| XuefuZhuyu Decoction | Taoren (Prunus persica)  Honghua (Carthamus tinctorius)  Danggui (Angelica sinensis)  Shengdihuang (Rehmannia glutinosa)  Niuxi (Achyranthes bidentata)  Chuanxiong (Ligusticum sinense )  Jiegeng (Platycodon grandiflorus)  Shaoyao (Paeonia lactiflora)  Zhike (Citrus aurantium)  Gancao (Glycyrrhiza uralensis)  Chaihu (Bupleurum chinense) | Rosaceae  Asteraceae  Apiaceae  Orobanchaceae  Amaranthaceae  Apiaceae  Campanulaceae  Paeoniaceae  Rutaceae  Apiaceae  Apiaceae |
| LiuweiDihuang Pill | Shudihuang (Rehmannia glutinosa)  Shanyurou (Tetradium ruticarpum))  Shanyao (Dioscorea polystachya)  Mudanpi (Paeonia suffruticosa)  Fulin (Poria Cocos)  Zexie (Alisma plantago-aquatica) | Orobanchaceae  Rutaceae  Dioscoreaceae  Paeoniaceae  Polyporaceae  Alismataceae |
| Ganluyin | Fulin (Poria Cocos)  Zexie (Alisma plantago-aquatica)  Gancao (Glycyrrhiza uralensis)  Shigao (gypsum fibrosum)  Hanshuishi (/)  Baizhu (Atractylodes macrocephala )  Guizhi (Cinnamomum cassia)  Zhuling (Agaric Polyporus)  Huashi (/) | Polyporaceae  Alismataceae  Apiaceae  /  /  Asteraceae  Lauraceae  Polyporaceae  / |
| Jiawei Xiaoyao Powder | Danggui (Angelica sinensis)  Shaoyao (Paeonia lactiflora)  Baizhu (Atractylodes macrocephala)  Chaihu (Bupleurum chinense)  Mudanpi (Paeonia suffruticosa)  Zhizi (Gardenia jasminoides)  Gancao (Glycyrrhiza uralensis) | Apiaceae  Paeoniaceae  Asteraceae  Apiaceae  Paeoniaceae  Rubiaceae  Fabaceae |
| Chuanxiong Chatiao Powder | Chuanxiong (Ligusticum sinense)  Jingjie (Nepeta cataria)  Baizhi (Angelica dahurica)  Jiegeng (Platycodon grandiflorus)  Gancao (Glycyrrhiza uralensis)  Huangqin (Scutellaria baicalensis)  Chuanbeimu (Fritillaria cirrhosa)  Zhizi (Gardenia jasminoides) | Apiaceae  Lamiaceae  Apiaceae  Campanulaceae  Fabaceae  Lamiaceae  Liliaceae  Rubiaceae |
| Gegen Decoction | Gegen (Pueraria edulis)  Mahuang (Ephedra sinica)  Guizhi (Cinnamomum cassia)  Shengjiang (Zingiber officinale)  Gancao (Glycyrrhiza uralensis)  Shaoyao (Paeonia lactiflora) | Leguminosae  Ephedraceae  Lauraceae  Zingiberaceae  Leguminosae  Ranunculaceae |
| Yinqiao Powder | Lianqiao (Forsythia suspensa)  Jinyinhua (Lonicera japonica)  Jiegeng (Platycodongrandiflorus)  Bohe (Mentha canadensis)  Zhuye (Lophatherum gracile)  Gancao (Glycyrrhiza uralensis)  Jingjiesui (Nepeta cataria)  Dandouchi (fermented soya beans, Glycine max or Dumasiatruncata)  Niubangzi (Arctium lappa) | Oleaceae  Caprifoliaceae  Campanulaceae  Labiatae  Gramineae  Leguminosae  Labiatae  Leguminosae  Compositae |
| Maxing GanshiDecoction | Mahuang (Ephedra sinica)  Xingren (seed of Armeniaca vulgaris)  Gancao (Glycyrrhiza uralensis)  Shigao (gypsum fibrosum) | Ephedraceae  Rosaceae  Leguminosae  / |
| TianwangBuxin Dan | Renshen (Panax ginseng)  Fuling (Poriacocos)  Xuanshen (Scrophularianingpoensis)  Danshen (Salvia miltiorrhiza)  Jiegeng (Platycodongrandiflorus)  Yuanzhi (Polygala tenuifolia)  Danggui (Angelica sinensis)  Wuweizi (Schisandra chinensis)  Maimendong (Ophiopogon japonicus)  Baiziren (seed of Platycladusorientalis)  Suanzaoren (seed of Ziziphus jujuba var. spinosa)  Shengdihuang (Rehmanniaglutinosa) | Araliaceae  Polyporaceae  Scrophulariaceae  Labiatae  Campanulaceae  Polygalaceae  Umbelliferae  Magnoliaceae  Liliaceae  Cupressaceae  Rhamnaceae  Scrophulariaceae |
| LongdanXiegan Decoction | Longdancao (Gentiana scabra)  Zhizi(Gardenia jasminoides)  Huangqin (Scutellariabaicalensis)  Mutong (Akebia trifoliata)  Zexie (Alisma plantago-aquatica)  Cheqianzi (seed of Plantago asiatica)  Chaihu (Bupleurum chinense)  Gancao (Glycyrrhiza uralensis)  Danggui (Angelica sinensis)  Shengdihuang (Rehmanniaglutinosa) | Gentianaceae  Rubiaceae  Labiatae  Lardizabalaceae  Alismataceae  Plantaginaceae  Umbelliferae  Leguminosae  Umbelliferae  Scrophulariaceae |
| Banxiaxiexin Decoction | Banxia (Pinelliaternata)  Huanglian (Coptis chinensis)  Huangqin (Scutellariabaicalensis)  Ganjiang (Zingiber officinale)  Gancao (Glycyrrhiza uralensis)  Dazao (Ziziphus jujuba)  Renshen (Panax ginseng) | Araceae  Ranunculaceae  Labiatae  Zingiberaceae  Leguminosae  Rhamnaceae  Araliaceae |
| Pingwei Powder (Pill) | Cangzhu (Atractylodeslancea)  Houpo (Houpoea officinalis)  Chenpi (Citrus reticulata)  Gancao (Glycyrrhiza uralensis) | Compositae  Magnoliaceae  Rutaceae  Leguminosae |
| ZhibaiDihuang Pill | Zhimu (Anemarrhenaasphodeloides)  Shudihuang (Rehmanniaglutinosa)  Shanzhuyu (Cornus officinalis)  Shanyao (Dioscoreapolystachya)  Mudanpi (Paeonia suffruticosa)  Fuling (Poriacocos)  Zexie (Alisma plantago-aquatica) | Liliaceae  Scrophulariaceae  Cornaceae  Dioscoreaceae  Ranunculaceae  Polyporaceae  Alismataceae |
| MaziRen Pill | Huomaren (seed of Cannabis sativa)  Shaoyao (Paeonia lactiflora)  Zhishi (seed of Citrus sinensis)  Dahuang (Rheum palmatum)  Houpo (Houpoea officinalis)  Xingren (seed of Armeniaca vulgaris) | Moraceae  Ranunculaceae  Rutaceae  Polygonaceae  Magnoliaceae  Rosaceae |
| Zhigancao Decoction | Zhigancao (Glycyrrhiza uralensis)  Shengjiang (Zingiber officinale)  Guizhi (Cinnamomum cassia)  Renshen (Panax ginseng)  Shengdihuang (Rehmanniaglutinosa)  Ejiao (donkey-hide gelatin)  Maimendong (Ophiopogon japonicus)  Huomaren (seed of Cannabis sativa)  Dazao (Ziziphus jujuba) | Leguminosae  Zingiberaceae  Lauraceae  Araliaceae  Scrophulariaceae  /  Liliaceae  Moraceae  Rhamnaceae |
